# Comparative left ventricular mechanical deformation in acute apical variant stress cardiomyopathy and acute anterior myocardial infarction utilizing 2‐dimensional longitudinal strain imaging

**DOI:** 10.1111/echo.14675

**Published:** 2020-05-21

**Authors:** Mohamed Ahmed, Mayank Sardana, Somwail Rasla, Jorge Escobar, Josiah Bote, Aline Iskandar, Khanh‐Van Tran, Dennis A. Tighe, Timothy P. Fitzgibbons, Gerard P. Aurigemma

**Affiliations:** ^1^ Division of Cardiovascular Medicine Beth Israel Deaconess Medical Center Boston MA USA; ^2^ Division of Cardiovascular Medicine University of California San Francisco San Francisco CA USA; ^3^ Department of Medicine The Warren Alpert Medical School Brown University Providence RI USA; ^4^ Division of Cardiovascular Medicine University of Massachusetts Medical School Worcester MA USA

**Keywords:** longitudinal strain, myocardial infarction, speckle tracking echocardiography, stress cardiomyopathy, Takotsubo cardiomyopathy

## Abstract

**Aims:**

Despite three decades of study, it is still challenging to discriminate acute apical variant stress cardiomyopathy (AVSCM) from acute left anterior descending‐myocardial infarction (LAD‐MI) at the time of presentation. A biomarker or practical imaging modality that can differentiate these two entities is highly desirable. Our objective was to characterize left ventricular (LV) mechanical deformation using 2‐dimensional (2D) echocardiographic strain imaging in an attempt to discriminate AVSCM from LAD‐MI at presentation.

**Methods and Results:**

We studied 108 women (60 AVSCM, 48 ST segment elevation LAD‐MI). All underwent echocardiography within 48 hours of presentation. 2D longitudinal strain (LS) from an 18‐segment LV model was performed, with global LS (GLS) taken as the average of all 18 segments. GLS was abnormal, but did not differentiate AVSCM from LAD‐MI. Mean LS of the basal and mid‐anterior, basal, and mid‐anteroseptum segments were significantly lower in LAD‐MI vs AVSCM group (−14 ± 9% vs −20 ± 8%; −11 ± 7% vs −14 ± 6%; −9 ± 8% vs −14 ± 8%; −9 ± 7% vs −13 ± 5%, respectively, all *P* ≤ .05). Mean LS of the basal inferior and inferolateral segments was significantly higher in the LAD‐MI vs. AVSCM group (−19 ± 9% vs −13 ± 7%; −23 ± 11% vs −18 ± 7%, respectively, all *P* ≤ .05). Using ROC curve analysis, segmental strain ratio of average basal inferior and inferolateral segments LS to average mid‐ and basal anterior and anteroseptum segments LS of ≥1.58 was 90% specific for LAD‐MI [area under the curve (AUC) 0.87; *P* < .001].

**Conclusion:**

Longitudinal strain patterns are useful in discriminating AVSCM from LAD‐MI patients at presentation and may be valuable in stratifying patients for invasive evaluation.

## 1 INTRODUCTION

Stress cardiomyopathy (SCM), also known as “Takotsubo” cardiomyopathy, was initially described by Sato et al in 1990.^1^ The disease is characterized by transient systolic and diastolic left ventricular dysfunction with a variety of wall‐motion abnormalities.^2^, ^3^ Although SCM is classically described as “apical ballooning syndrome,” variants including mid‐ventricular, basal, or focal segmental have also been described.^4^ Apical variant SCM (AVSCM) is characterized by wall‐motion abnormalities in the apical and mid‐ventricular segments, a pattern virtually indistinguishable from myocardial dysfunction due to acute left anterior descending‐myocardial infarction (LAD‐MI). Despite extensive investigation of SCM over the past three decades, the pathophysiology is still not well understood, and it remains challenging to discriminate AVSCM from LAD‐MI in the acute phase. Due to lack of any point‐of‐care discriminating tests, AVSCM patients are triaged similar to LAD‐MI and often undergo emergent coronary angiography to rule out the presence of a culprit obstructive coronary lesion.

Assessment of myocardial deformation by two‐dimensional (2D) speckle tracking echocardiography (STE) provides a means to objectively quantify myocardial mechanical function.^5^, ^6^ Application of STE provides unique information on regional and global ventricular function with main areas of applications include cardiomyopathy, valvular, and ischemic heart disease.^5^ Over the past two decades, the addition of STE has refined the ability of echocardiography to differentiate active myocardial contraction from passive motion, which is often difficult visually. Our objective in this study was to characterize global and regional LV mechanical activation using 2D strain imaging in an attempt to arrive at a parameter or cluster of parameters that might distinguish AVSCM from LAD‐MI at presentation.

## 2 METHODS

The study was approved by University of Massachusetts Institutional Review Board.

### 2.1 Study cohort

A total of 60 consecutive women who were diagnosed with acute AVSCM at the University of Massachusetts Medical Center from 2007 to 2014 were retrospectively identified. Electronic medical records were reviewed to confirm the presence of SCM by Mayo Clinic diagnostic criteria^7^ as follows: the presence of a transient abnormality in left ventricular wall motion beyond a single epicardial coronary artery perfusion territory, the absence of obstructive coronary artery disease or angiographic evidence of acute plaque rupture, the presence of new electrocardiographic abnormalities or elevation in cardiac troponin levels, and the absence of pheochromocytoma and myocarditis. Similarly, 48 consecutive women with ST segment elevation LAD‐MI were selected by searching the catheterization laboratory database who presented from 2007 to 2014. Demographic, clinical, and laboratory characteristics were abstracted from the electronic medical record by trained study staff.

### 2.2 Echocardiographic and strain analysis

**FIGURE 1:**
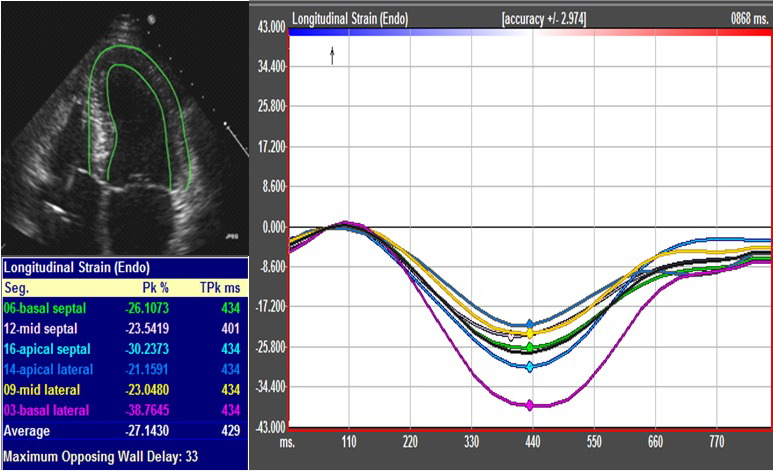
Longitudinal strain curves from left ventricle (LV) apical four‐chamber view in a normal subject using speckle tracking echocardiography. Endocardial borders were traced, and myocardial walls were tracked. The interactive software then automatically tracked myocardial motion during the entire LV mechanical cycle and divided each image into six segments. LV was divided into 18 segments with six segments per view, and global longitudinal strain (GLS) was averaged over all the trackable segments

Each participant underwent a clinically indicated transthoracic echocardiogram within 48 hours of the coronary angiogram. Echocardiography was performed with a commercially available standard ultrasound scanner using Vivid 7 (GE) and Phillips IE‐33 machines. Images were analyzed by two experienced investigators blinded to the clinical data. Left ventricular (LV) ejection fraction (EF) was calculated using by Simpson's method, and LV dimensions were measured by the methods recommended by the American Society of Echocardiography (ASE) guidelines.^8^ For 2D STE, analysis was performed on digital gray scale images (60–100 frames per second) of the standard apical two‐, three‐, and four‐chamber views using 18‐segment LV model, as reported previously.^9^ Two experienced operators (MA and MS) performed the strain analysis and were blinded to the study groups. Commercial software (2D Cardiac Performance Analysis v1.1.3 TomTec Image Systems) was used to analyze images, with endocardial regions of interest placed and myocardial walls tracked. The interactive software then automatically tracked myocardial motion during the entire LV mechanical cycle and divided each image into six segments. LV was divided into 18 segments with 6 segments per view, and global longitudinal strain (GLS) was averaged over all the trackable segments (Figure 1). We then calculated segmental strain ratio by dividing the average of strain values in the non‐LAD territory segments [basal inferior (b Inferior) and basal inferolateral (b Inferolateral) segments] by the average strain values in the LAD territory segments [basal anterior (b Anterior), mid‐anterior (m Anterior), basal anteroseptal (b AS) and mid‐anteroseptal (m AS) segments]. A version of the formula is as below:

$$\text{Segmental}\;\text{strain}\;\text{ratio}=\frac{b\;\text{Inferior}+b\;\text{inferolateral}}{b\;\text{Anterior}+b\;\text{AS}+m\;\text{Anterior}+m\;\text{AS}}$$

The time to peak strain was measured from the beginning of the QRS complex on the electrocardiogram (ECG), and the standard deviation of the 18 different time intervals to peak longitudinal strain in each participant was calculated to quantify LV mechanical dispersion reflecting myocardial contraction heterogeneity as previously described.^10^, ^11^ Segments with only positive strain, as in dyskinetic segments, and segments with strain curves oscillating around the zero line, as in akinetic segments, were not included in time measurements. Of note, we did not include the mid‐inferior and inferolateral segments in the segmental strain ratio for two reasons, (a) in patients with wrap‐around LAD, a significant proportion of mid‐inferior wall may partly be supplied by LAD and (b) mid‐segments are affected in a minor subset SCM (except in mid‐cavity variant of SCM). This approach is further supported by the lack of significant differences in segmental strain values in mid‐inferior and mid‐inferolateral segments in SCM and LAD‐MI groups [mid‐inferior (%) −10 ± 5 in SCM vs −10 ± 6 in LAD‐MI group (*P* = .85); mid‐inferolateral (%) −13 ± 6 in SCM vs −13 ± 7 LAD‐MI group (*P* = .88)].

### 2.3 Statistical methods

Continuous data are presented as mean ± standard deviation (SD) unless otherwise specified. Comparisons of continuous variables were performed by using t tests, and comparisons of categorical variables were performed by using the chi‐square (χ^2^) or Fisher's exact test, as appropriate. For mechanical dispersion (SD of time to peak longitudinal strain in all 18 segments), statistical difference was determined by using the F test. Receiver operating characteristic (ROC) curve analysis was performed to identify the best cutoff values for continuous variables predicting LAD‐MI. We further performed prespecified stratified sensitivity analyses in subgroups based on the location of LAD lesion (ie, proximal LAD vs AVSCM and mid‐ or distal LAD vs AVSCM), and absence or presence of nonculprit coronary artery lesions (more than 50% stenosis). Performance of the segmental strain ratio cutoff identified from the general sample was measured using ROC curve analysis in these subgroups. Statistical significance for all tests was defined as *P* value ≤ 0.05.

## 3 RESULTS

Of the total 108 female participants, 60 patients had acute AVSCM and 48 patients had ST segment elevation LAD‐MI. Baseline characteristics of study participants are depicted in Table 1. Of the 48 patients with LAD‐MI, 56% had proximal LAD involvement and 44% had mid‐ or distal LAD involvement. Compared to LAD‐MI participants, AVSCM participants were older (69 ± 12 vs 62 ± 15, *P* = .009) and less likely to have diabetes mellitus (12% vs 29%, *P* = .04). ST segment elevation was present in 33% of the AVSCM patients on presentation, and corrected QT interval was significantly prolonged when compared to LAD‐MI group (490 ± 45 vs 466 ± 37 ms, *P* = .004). This is consistent with previous findings that patients with AVSCM have significant QT prolongation and are at risk of Torsades de pointes and ventricular fibrillation.^12^, ^13^ Peak cardiac troponin was significantly higher in the LAD‐MI group (troponin I 64 ± 55 µg/L vs 3.2 ± 4.6 µg/L, *P* < .001). On presentation, average heart rate and systolic blood pressure were similar between the two groups. LVEF was equally reduced in both groups (37 ± 12% for AVSCM vs 38 ± 12% for LAD‐MI). In LAD‐MI group, nonculprit coronary artery lesions were present in a third of the participants (more than 50% stenosis; right coronary artery involvement in 15 participants, circumflex artery involvement in 7 participants).

**TABLE 1:** Baseline clinical and echocardiographic characteristics

Characteristics	AVSCM (n = 60)	LAD‐MI (n = 48)	*P* value
Age, y	69 ± 12	62 ± 15	.009
Female, %	100	100	1.00
Hypertension, %	72	58	.18
Diabetes mellitus, %	12	29	.04
Systolic blood pressure, mm Hg	121 ± 20	119 ± 16	.58
Heart rate, beats/min	82 ± 19	83 ± 18	.78
ECG on presentation			
ST elevation, %	33	100	<.0001
T‐wave inversion, %	53	48	.74
Corrected QT interval, ms	490 ± 45	466 ± 37	.004
Peak troponin I, µg/L	3.2 ± 4.6	64 ± 55	<.0001
LVEF, %	37 ± 12	38 ± 12	.67
LVIDd, mm	45 ± 7	45 ± 5	1.00
LVIDs, mm	29 ± 7	32 ± 8	.04
LVEDV^a^, mL	80 ± 27	90 ± 28	.33
LVESV^a^, mL	43 ± 15	51 ± 25	.23

Note

Data shown are mean ± SD for continuous variables and percent for categorical values. Statistical differences for continuous variables were determined by using t test. Statistical differences between categorical data were determined with chi‐square test.

Abbreviations: AVSCM = apical variant stress cardiomyopathy; ECG = electrocardiogram; LAD‐MI = left anterior descending coronary artery‐myocardial infarction; LVEDV = left ventricular end‐diastolic volume; LVEDV = left ventricular end‐systolic volume; LVEF = left ventricular ejection fraction; LVIDd = left ventricular internal dimension = diastole; LVIDs = left ventricular internal dimension, systole.

^a^ Data were characterized from a subset of patients (N = 38)

### 3.1 Feasibility and reliability of strain measurements

Overall analysis was possible in 1656 out of 1944 segments providing a feasibility of 85% for the study. The inter‐ and intra‐observer variability in measurement of global and segmental longitudinal strain in our laboratory has previously been reported at 12.2% and 10.7%, respectively.^14^

### 3.2 LV Global and segmental longitudinal strain

**FIGURE 2:**
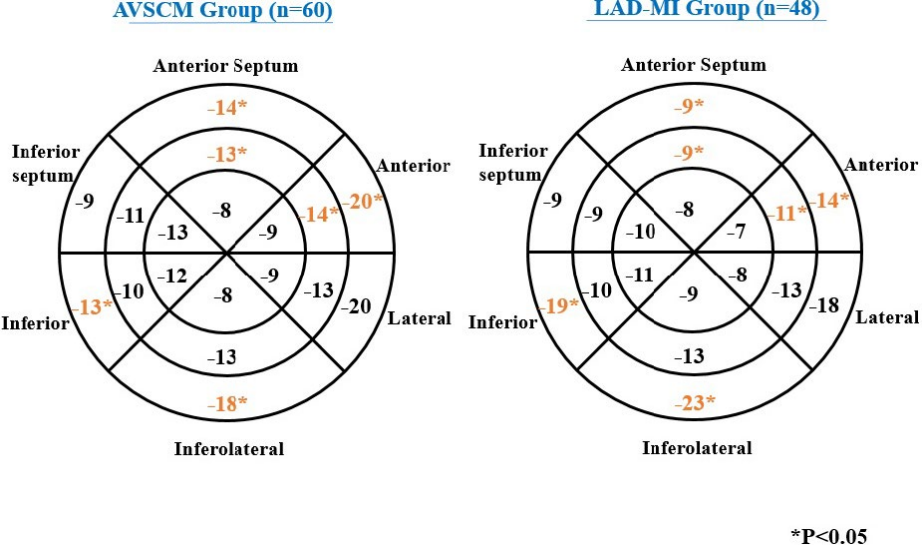
Bull's‐eye diagram of 18‐segment left ventricular (LV) model representing mean longitudinal strain values in two groups. When compared to apical variant stress cardiomyopathy (AVSCM) group, left anterior descending coronary artery‐myocardial infarction group (LAD‐MI) group had lower segmental strain values in the LAD territory and higher segmental strain values in the non‐LAD territory segments

**FIGURE 3:**
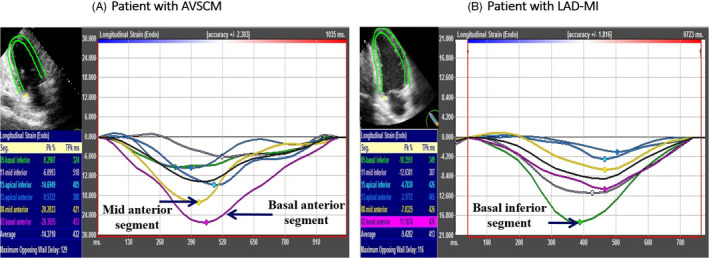
Example of longitudinal strain curves from left ventricle (LV) apical two‐chamber view in a patient with apical variant stress cardiomyopathy (AVSCM) (A) and a patient with left anterior descending coronary artery‐myocardial infarction (LAD‐MI) (B). In patient B, longitudinal strain values were lower in the basal and mid‐anterior segments when compared to patient A. On the contrary, longitudinal strain in the basal inferior segment is higher in patient B compared to the patient A

GLS was significantly but equally reduced between the AVSCM and LAD‐MI groups (−13 ± 4% vs −12 ± 5%, *P* = .32). Table 2 shows a comparison of GLS and mean segmental LS values between AVSCM and LAD‐MI groups. Although mean segmental LS was reduced in nearly all segments, the apical segments were more affected in both groups. When compared to the AVSCM group, the LAD‐MI group had significantly lower average LS values in the LAD territory segments (−14 ± 9% vs −20 ± 8% for the basal anterior, −11 ± 7% vs −14 ± 6% for the mid‐anterior, −9 ± 8% vs −14 ± 8% for the basal anteroseptum, and −9 ± 7% vs −13 ± 5% for the mid‐anteroseptum, *P* ≤ .05 for all comparisons; Figure 2). On the contrary, the average LS in the non‐LAD territory segments was significantly higher in LAD‐MI when compared to the AVSCM group (−19 ± 9% vs −13 ± 7% for basal inferior and −23 ± 11% vs −18 ± 7% for basal inferolateral segments, *P* ≤ .05 for both comparisons; Figure 2). An example of segmental LS comparison between a patient with LAD‐MI and a patient with AVSCM from apical two‐chamber view is shown in Figure 3.

**TABLE 2:** Global and segmental longitudinal strain between two groups

	AVSCM (n = 60)	LAD‐MI (n = 48)	*P* value
Longitudinal strain; four‐chamber view			
Basal septal (%)	−9 ± 6	−9 ± 7	.90
Mid‐septal (%)	−11 ± 6	−9 ± 6	.08
Apical septal (%)	−13 ± 8	−10 ± 7	.10
Apical lateral (%)	−9 ± 7	−8 ± 7	.81
Mid‐lateral (%)	−13 ± 4	−13 ± 7	.79
Basal lateral (%)	−20 ± 7	−18 ± 9	.12
Longitudinal strain; two‐chamber view			
Basal inferior (%)	−13 ± 7	−19 ± 9	.0004
Mid‐inferior (%)	−10 ± 5	−10 ± 6	.85
Apical inferior (%)	−12 ± 10	−11 ± 9	.71
Apical anterior (%)	−9 ± 8	−7 ± 6	.11
Mid‐anterior (%)	−14 ± 6	−11 ± 7	.01
Basal anterior (%)	−20 ± 8	−14 + 9	.002
Longitudinal strain; three‐chamber view			
Basal inferolateral (%)	−18 ± 7	−23 ± 11	.01
Mid‐inferolateral (%)	−13 ± 6	−13 ± 7	.88
Apical inferolateral (%)	−8 ± 7	−9 ± 8	.72
Apical anteroseptal (%)	−8 ± 7	−8 ± 7	.75
Mid‐anteroseptal (%)	−13 ± 5	−9 ± 7	.005
Basal anteroseptal (%)	−14 ± 8	−9 ± 8	.006
GLS (%)	−13 ± 4	−12 + 5	.32
Mechanical dispersion (ms)	95	84	.38

Note

Data shown are mean ± SD. Statistical differences were determined by using the *t* test. For mechanical dispersion (SD of time to peak longitudinal strain in all 18 segments), statistical difference was determined by using the F test.

Abbreviations: AVSCM = apical variant stress cardiomyopathy; GLS = global longitudinal strain; LAD‐MI = left anterior descending coronary artery‐myocardial infarction.

**FIGURE 4:**
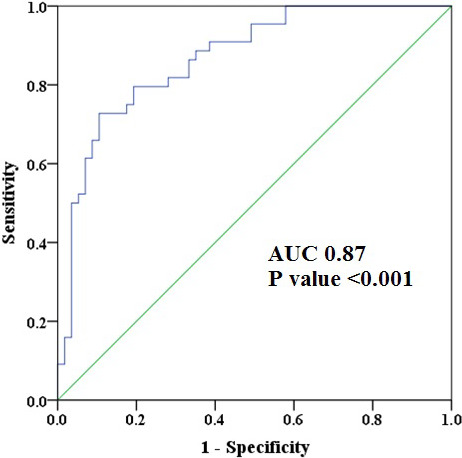
Receiver operating characteristic (ROC) curve for the association of segmental strain ratio and left anterior descending coronary artery‐myocardial infarction (LAD‐MI). Segmental strain ratio of ≥1.58 is 90% specific for prediction of LAD‐MI with area under the curve (AUC) of 0.87

Median segmental strain ratio in the LAD‐MI group was 2.1 (interquartile range of 1.3–3.6) and 1.0 (interquartile range of 0.6–1.5) in the AVSCM group. Using ROC curve analysis, segmental strain ratio of ≥1.58 was 90% specific and 73% sensitive for LAD‐MI (AUC 0.87, 95% CI 0.80–0.94; *P* < .001; Figure 4, Table 3) Mechanical dispersion was abnormal but equally prolonged between the two groups (93 msec for AVSCM vs 84 msec for LAD‐MI, *P* = .38).

**TABLE 3:** Association of segmental strain ratio and left anterior descending artery‐myocardial infarction (LAD‐MI) in various subgroups

MI participants included	AUC	95% Confidence interval	Segmental strain ratio ≥ 1.58	
			Sensitivity (%)	Specificity (%)
All LAD‐MI participants (N = 44)^a^ vs AVSCM	0.87	0.80–0.94	73	90
LAD disease location				
Proximal LAD (N = 22) vs AVSCM	0.88	0.80–0.96	73	90
Mid‐ to distal LAD (N = 22) vs AVSCM	0.86	0.77–0.95	73	90
Nonculprit artery lesions				
Absent (N = 30) vs AVSCM	0.89	0.82–0.96	77	90
Present (N = 14) vs AVSCM	0.82	0.70–0.94	64	90

Note

Abbreviations: AUC = area under the curve; AVSCM = apical variant stress cardiomyopathy.

^a^ Segmental strain ratio could not be calculated in 4 of 48 participants in the left anterior descending‐myocardial infarction (LAD‐MI) group due to poor tracking of one or more segments required for calculation of ratio (basal inferior, basal inferolateral, basal anteroseptal, mid‐anteroseptal, basal anterior, and mid‐anterior segments).

### 3.3 Sensitivity analyses

In the first set of sensitivity analyses, we measured the association of segmental strain ratio with LAD‐MI in prespecified stratified subgroups based on the location of LAD lesion using ROC curve analysis (Table 3). Segmental strain ratio ≥1.58 had similar sensitivity and specificity in discriminating proximal LAD‐MI from AVSCM as mid‐ or distal LAD‐MI from AVSCM (sensitivity 73% and specificity 90% for both). In the second set of sensitivity analyses, we compared the performance of segmental strain ratio in discriminating LAD‐MI from AVSCM in subgroups developed based on the absence or presence of nonculprit coronary artery lesions (in right coronary artery and/or circumflex artery territories, stenosis more than 50%). Segmental strain ratio ≥1.58 had lower sensitivity for the participants with nonculprit coronary artery lesions (sensitivity 64%, specificity 90%, AUC: 0.82) than in the participants without nonculprit artery lesions (sensitivity 77%, specificity 90%, AUC: 0.89; Table 3).

## 4 DISCUSSION

In this cross‐sectional study of 108 female participants, we have demonstrated significant differences in segmental LS pattern between AVSCM and acute LAD‐MI. Due to the differences in the segmental strain between the two groups, we were able to develop as a simple segmental strain ratio which has the potential to be used as a point‐of‐care tool to discriminate these two entities in the acute care settings.

### 4.1 Clinical presentation of stress cardiomyopathy and acute myocardial infarction

SCM, initially recognized in postmenopausal females with emotional triggers, has now been associated with a variety of physical illnesses.^4^ However, female gender remains the strongest risk factor and nearly 90% of SCM patients are women.^4^, ^15^, ^16^ To ensure uniformity in our cohort, we only selected female participants with AVSCM since strain parameters might potentially be influenced by patient's gender among other factors.^17^, ^18^ All participants in our study had presented with chest pain and/or dyspnea and had ECG changes suggestive of myocardial ischemia. LV systolic dysfunction on echocardiogram was similar on presentations between the two groups. Due to such degree of clinical similarity, differentiation of SCM from myocardial infarction was not possible on presentation and all participants underwent coronary angiography.

### 4.2 Speckle tracking echocardiography and longitudinal strain measurement

Assessment of myocardial deformation by STE provides means to objectively quantify regional and global myocardial mechanical function.^5^, ^6^ Four STE‐based main approaches have been used, longitudinal, radial, circumferential, and transverse strain.^5^, ^6^ When compared to other approaches, LS approach has shown excellent accuracy with reliable inter‐observer and intra‐observer reproducibility.^19^, ^20^ Moreover, LS measurement has been shown to be consistent irrespective of the level of operator experience.^21^

### 4.3 Longitudinal strain in stress cardiomyopathy and myocardial infarction

In AVSCM and LAD‐MI, we found diminished mean LS values in nearly all segments when compared to normal values.^17^ In addition, segmental variability was evident, as the LAD‐MI group had significantly lower longitudinal strain in the basal to mid‐anterior and anteroseptal segments in comparison with the AVSCM group. As expected, the non‐LAD territory segments, basal inferior and inferolateral segments, were relatively spared, perhaps, due to greater compensatory hyperkinesis in the non‐LAD perfusion beds. We also showed that the calculated median segmental strain ratio was different between the two groups. ROC curve analysis revealed segmental strain ratio of ≥1.58 to be a powerful predictor of the diagnosis of LAD‐MI on presentation with 90% specificity. In the AVSCM group, LS was uniformly reduced corresponding to global myocardial disease extending beyond single epicardial coronary distribution and, possibly, global myocardial stunning.^22^ In our prespecified sensitivity analyses, we further demonstrated that the performance of segmental strain ratio in discriminating LAD‐MI from AVSCM was not affected by the location of LAD lesion. Although the presence of nonculprit coronary artery lesions led to slightly lower sensitivity of segmental strain ratio ≥1.58 in discriminating LAD‐MI from AVSCM, the overall performance of the model was comparable to the general cohort (AUC 0.82, specificity 0.90).

Prior investigators have reported LS differences between AVSCM and LAD‐MI groups in smaller cohorts. Although the results are variable, all have concluded segmental strain differences exist between the two groups.^23^, ^24^, ^25^ Heggermann et al measured segmental LS in 24 participants, 12 with LAD‐MI, and 12 with AVSCM.^23^ In concordance with our results, they observed significantly higher LS in non‐LAD territory segments in the LAD‐MI group. The difference was predominantly due to higher mean LS values in the inferior, inferolateral segments, and relative preservation of the mid‐lateral segment. In that study, no significant difference was reported in the anterior and anteroseptal segments between the AVSCM and LAD‐MI groups. This could be explained by the small sample size and the fact that the study groups were not sex‐matched.

Mansencal et al compared mean LS values between 14 AVSCM patients and 14 chronic LAD occlusion patients using velocity vector imaging.^25^ In the LAD occlusion group, they observed lower LS in the basal septal segments compared to the basal lateral segments. These segmental differences were not detected in the AVSCM group. No information was given on the anteroseptal and inferolateral segments as the LV apical three‐chamber views were not analyzed. Briasoulis et al recently described regional and global LS in 24 SCM patients (apical and mid‐ventricular variants) and 24 coronary artery disease (CAD) patients (LAD and multivessel CAD).^24^ Mean basal segment strain was significantly higher in both SCM variants compared to the CAD group, although no specific segmental analysis was reported. In agreement with our results, GLS was similarly reduced in the two groups. Although our results support their conclusion that the pattern of longitudinal strain in SCM is different from LAD‐MI, our findings cannot be directly compared to their observations due to mixed patient population, lack of sex‐match, and more importantly, the lack of specific segmental analysis.

With inclusion of larger cohorts, we were able to detect smaller differences in LS patterns which might not have been detected in the prior studies due to small sample size, lack of sex‐match, or lack of specific segmental analysis. Segmental strain ratio was calculated based on the difference in strain patterns in AVSCM and LAD‐MI. This novel ratio discriminated LAD‐MI from AVSCM in the acute settings with great accuracy.

### 4.4 LV GLS and mechanical dispersion

Although GLS was lower compared to the normal values,^5^ we found no significant difference between the LAD‐MI and AVSCM groups. In an attempt to derive a parameter that differentiates the two groups upon presentation, we quantified LV mechanical dispersion as it reflects myocardial contraction heterogeneity**.** LV mechanical dispersion has been shown to predict ventricular arrhythmia in patients after MI.^10^, ^11^ Haugaa et al reported significantly higher arrhythmia rates in patients with MI and mechanical dispersion of >70 ms when compared to those with mechanical dispersion of <70 ms In our study, although mechanical dispersion was not significantly different between the two groups, it was remarkably prolonged in both groups when compared to the previously reported values (Table 2).^10^, ^11^ This difference is possibly due to the fact that our analysis was conducted in acute presentation as opposed to prior reports where the measurements performed postimplantable cardioverter–defibrillator (ICD) implantation.

## 5 LIMITATIONS

Our data have certain important limitations. First, our sample size is relatively small although, to our knowledge, the current cohort is the largest cohort comparing the differences in myocardial strain between the AVSCM and LAD‐MI patients. Our findings are thus sufficient to form a hypothesis, and verification in a larger cohort is required. Another limitation is that all LAD‐MI patients had echocardiograms performed following revascularization and might have had partial recovery of longitudinal strain in the LAD territory segments. This, however, is unlikely given the short period of time between LAD revascularization and obtaining echocardiograms (usually <24 hours in our institution for ST segment elevation MI). Moreover, the segmental strain ratio will likely be higher for echocardiograms performed prior to revascularization and thus increasing difference between the two groups. Another limitation is the absence of B‐type natriuretic peptide (BNP) measurements for most of our participants. In a retrospective cohort analysis, Randhawa et al found BNP to be significantly elevated in SCM patients when compared to MI patients.^26^ Future studies should test the incremental value of segmental strain ratio when added to BNP measurement as it might further discriminate AVSCM from LAD‐MI patients. Finally, significant variations in LS have been reported between various commercial softwares.^17^ The segmental strain ratio, however, should remain stable between the softwares.

## 6 CONCLUSIONS

In this cross‐sectional study, we have demonstrated significant differences in segmental LS pattern between acute AVSCM and LAD‐MI. To our knowledge, this is the largest cohort comparison aimed at identifying the differences in myocardial strain between the two groups. We, for the first time, describe a segmental strain ratio which can discriminate between these two entities with high sensitivity and specificity. This novel ratio has the potential to be used in stratifying patients for invasive studies in the acute care settings. Future prospective validation studies are warranted in larger cohorts.

## CONFLICT OF INTEREST

None.

## Supporting information


**Figure S1.** Receiver‐operating characteristic (ROC) curve for the association of segmental strain ratio and myocardial infarction based on the location of LAD lesion. Segmental strain ratio of ≥1.58 is 90% specific for prediction of LAD‐MI in both subgroups with area under the curve (AUC) of 0.88 for proximal‐LAD and 0.86 for mid‐LAD subgroup.
**Figure S2.** Receiver‐operating characteristic (ROC) curve for the association of segmental strain ratio and left anterior descending coronary artery myocardial infarction (LAD‐MI) in subgroups based on absence of non‐culprit coronary artery lesions. Segmental strain ratio of ≥1.58 is 90% specific for prediction of LAD‐MI in both subgroups with area under the curve (AUC) of 0.89 for those without non‐culprit coronary artery lesions and 0.82 for those with non‐culprit coronary artery lesions.
