# Supplementary material for: Comparative left ventricular mechanical deformation in acute apical variant stress cardiomyopathy and acute anterior myocardial infarction utilizing 2‐dimensional longitudinal strain imaging
Source: Echocardiography. 2020 May 21;37(6):832–40. doi: 10.1111/echo.14675 (PMC7383586; doi:10.1111/echo.14675)

Figure 1

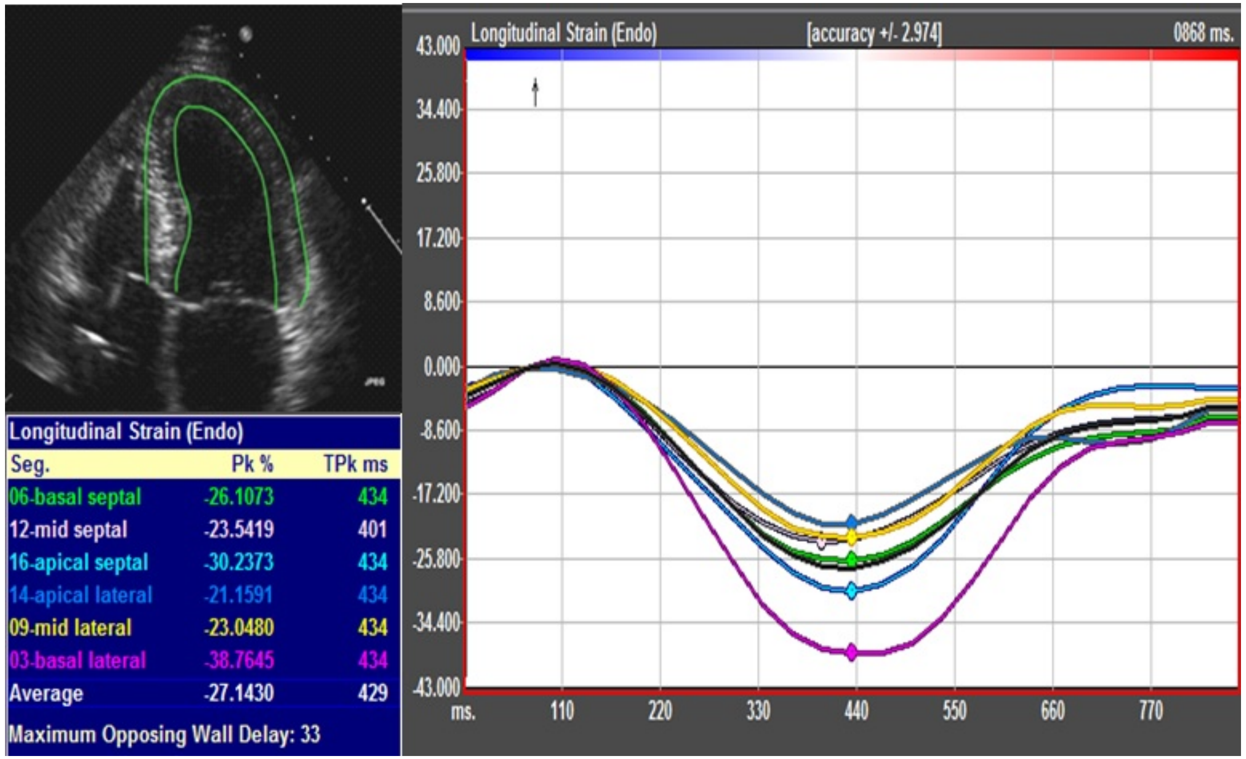
Longitudinal Strain (Endo)
Seg. Pk % TPk ms
06-basal septal -26.1073 434
12-mid septal -23.5419 401
16-apical septal -30.2373 434
14-apical lateral -21.1591 434
09-mid lateral -23.0480 434
03-basal lateral -38.7645 434
Average -27.1430 429
Maximum Opposing Wall Delay: 33
Longitudinal Strain (Endo)
[accuracy +/- 2.974]
0868 ms.
43.000
34.400
25.800
17.200
8.600
0.000
-8.600
-17.200
-25.800
-34.400
-43.000
ms.
110
220
330
440
550
660
770

Figure 2

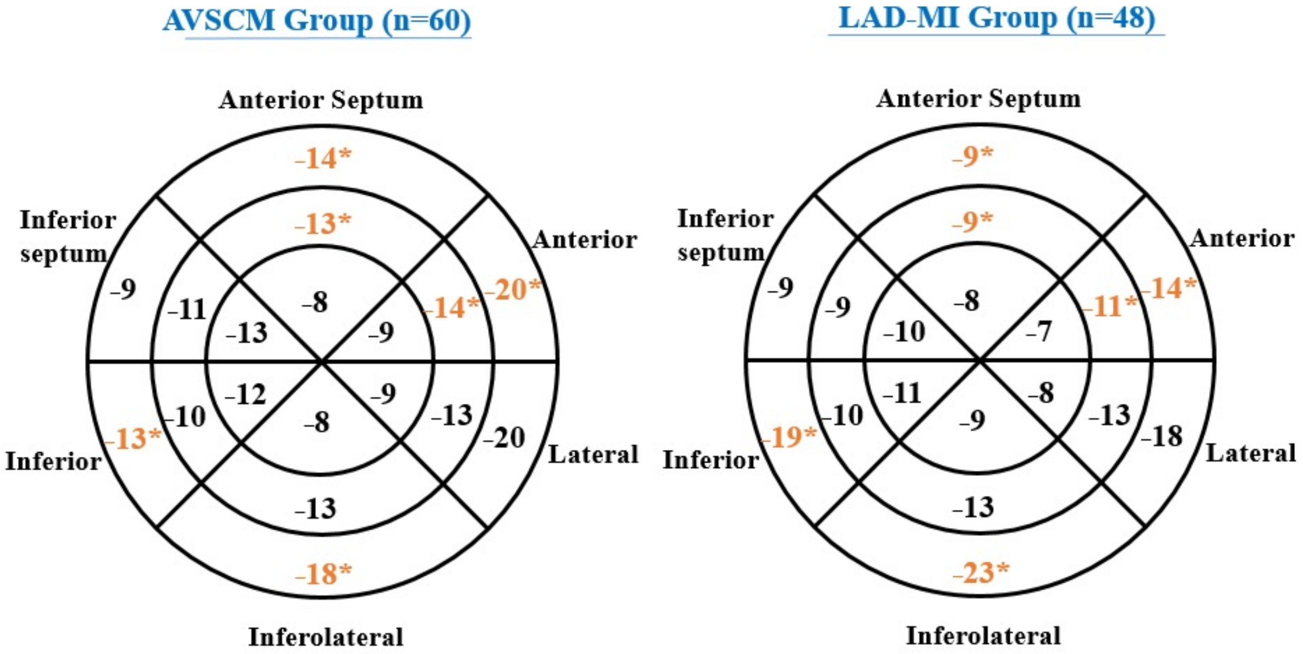

*P<0.05

Figure 3

A. Patient with AVSCM

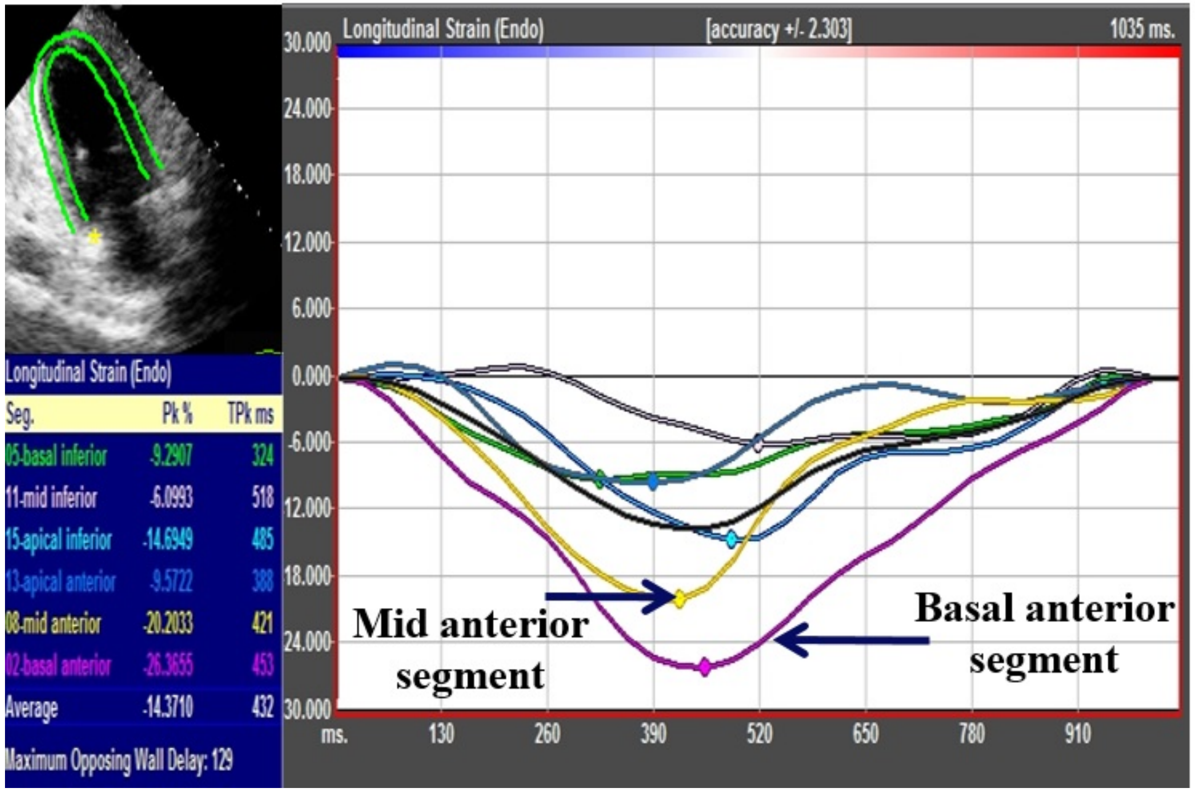

B. Patient with LAD-MI

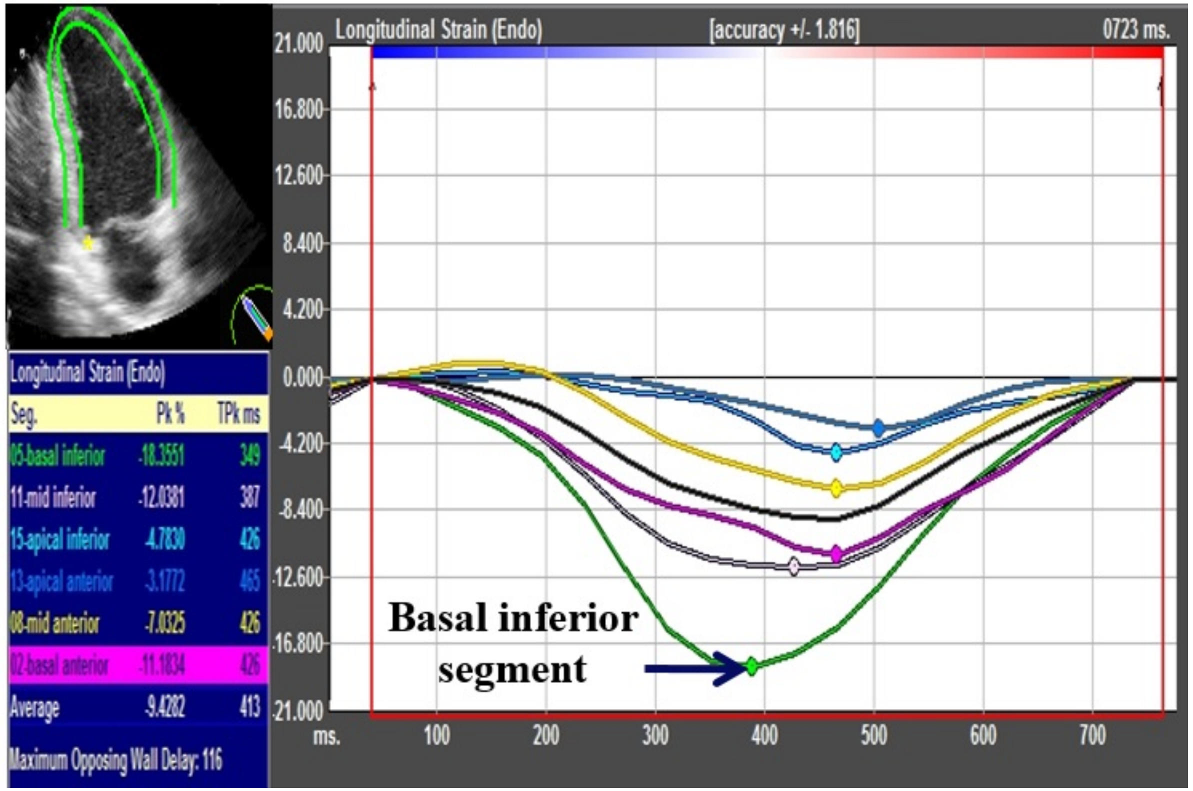

Figure 4

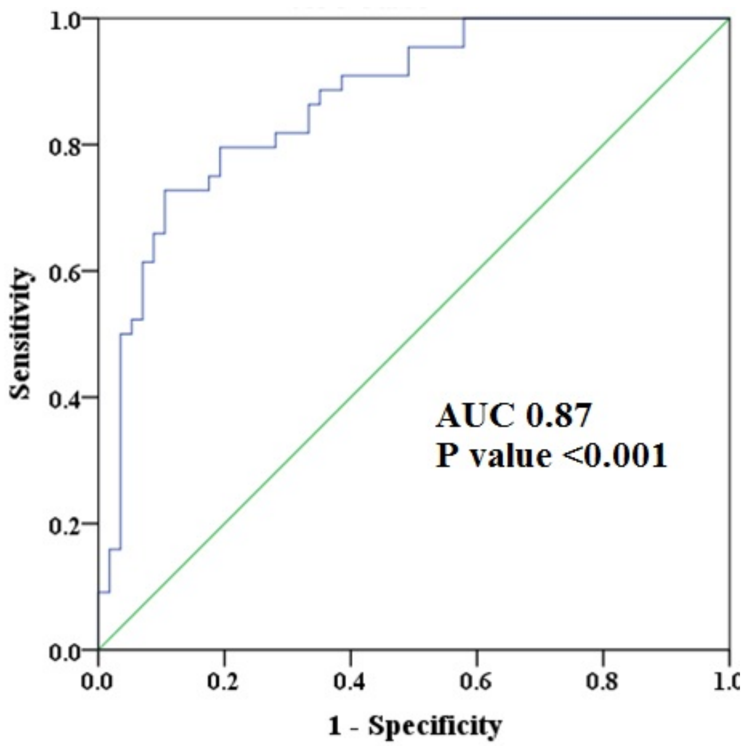
1.0
0.8
0.6
0.4
0.2
0.0
Sensitivity
AUC 0.87
P value <0.001
0.0
0.2
0.4
0.6
0.8
1.0
1 - Specificity

Supplemental Figure 1

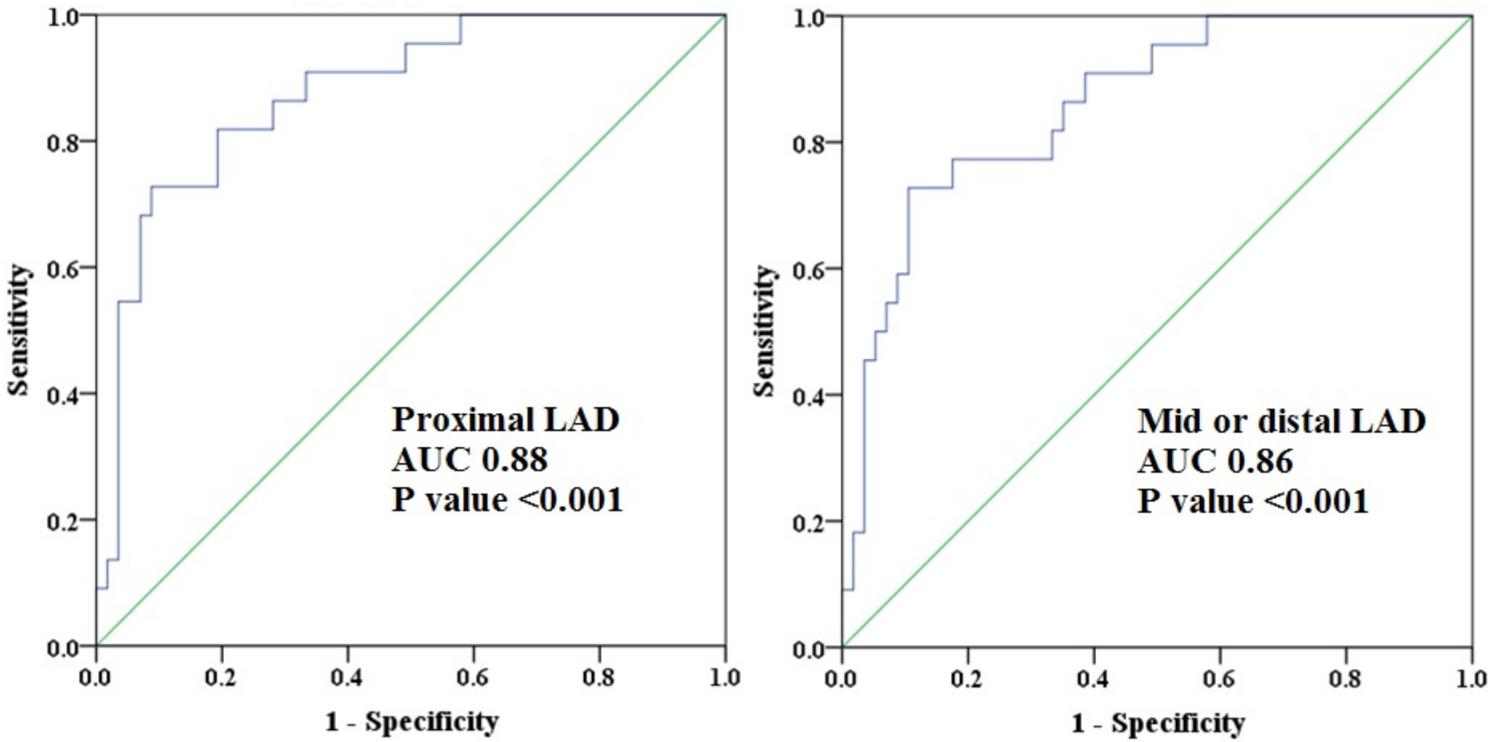

Supplemental Figure 2

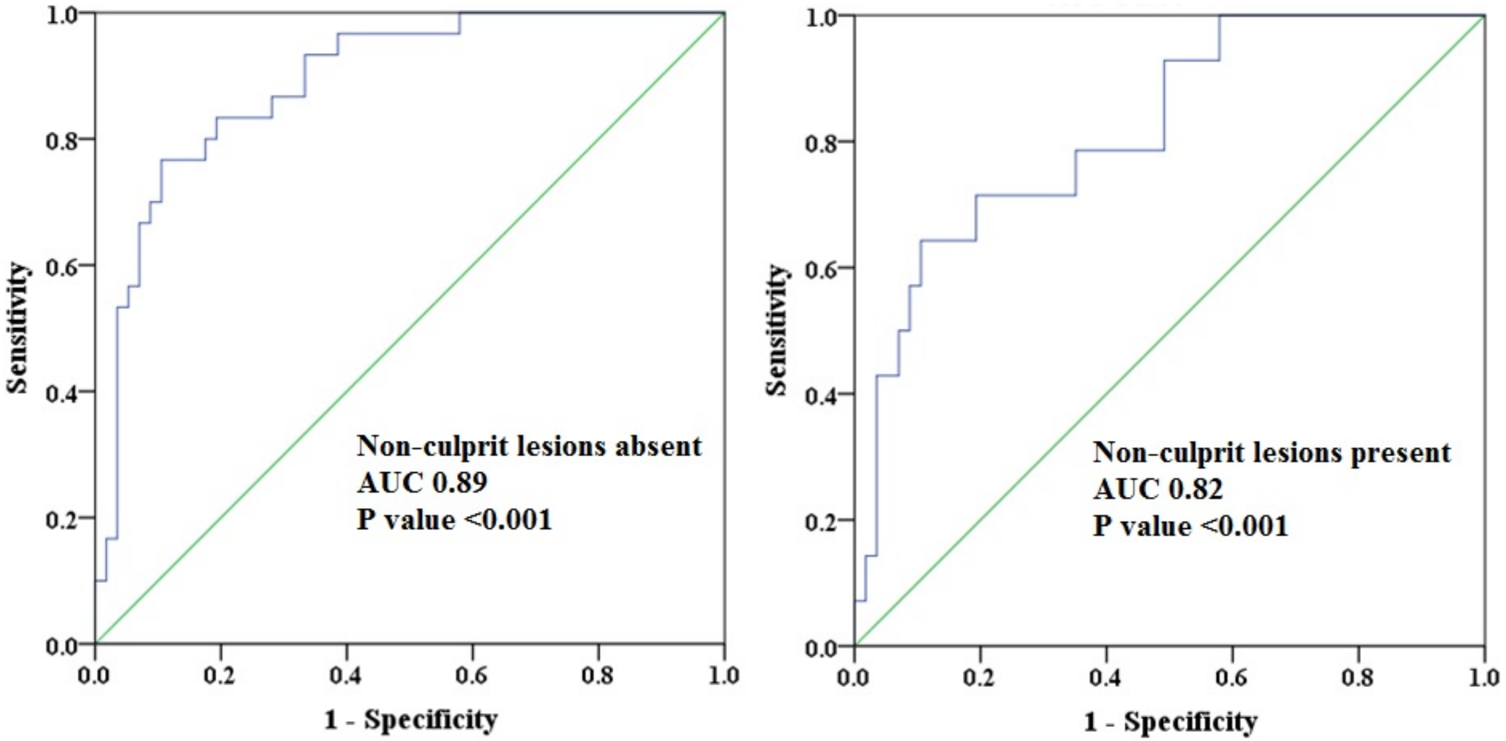

Supplement: Supplementary file 1 — Figure S1. Receiver‐operating characteristic (ROC) curve for the association of segmental strain ratio and myocardial infarction based on the location of LAD lesion. Segmental strain ratio of ≥1.58 is 90% specific for prediction of LAD‐MI in both subgroups with area under the curve (AUC) of 0.88 for proximal‐LAD and 0.86 for mid‐LAD subgroup. Figure S2. Receiver‐operating characteristic (ROC) curve for the association of segmental strain ratio and left anterior descending coronary artery myocardial infarction (LAD‐MI) in subgroups based on absence of non‐culprit coronary artery lesions. Segmental strain ratio of ≥1.58 is 90% specific for prediction of LAD‐MI in both subgroups with area under the curve (AUC) of 0.89 for those without non‐culprit coronary artery lesions and 0.82 for those with non‐culprit coronary artery lesions. [file ECHO-37-832-s001.pdf]
